# Inheritance patterns and heterosis of key floral traits in interspecific hybrids of *Clematis tientaiensis* × *C. lanuginosa*: implications for breeding compact cultivars

**DOI:** 10.3389/fpls.2026.1792549

**Published:** 2026-04-22

**Authors:** Xinli Zeng, Dujuan Shi, Shunyun He, Duojie Wei, Zhigao Liu

**Affiliations:** 1 School of Landscape Architecture, Zhejiang Agriculture and Forestry University, Hangzhou, Zhejiang, China; 2 Institute of Zhoushan Forestry Academy of Zhejiang, Zhoushan, Zhejiang, China

**Keywords:** *Clematis*, heterosis, hybrid breeding, mixed genetic analysis, phenotypic trait inheritance

## Abstract

*Clematis* cultivars are predominantly developed through hybridization, yet the genetic architecture of key ornamental traits remains poorly understood. In this study, *C. lanuginosa* and *C. tientaiensis*, along with their F_1_ progeny, were utilized to investigate the inheritance patterns of ten floral and vegetative traits. Specifically, we analyzed flower stalk length (FS), flower diameter (FD), sepal length (CL), sepal width (CW), stamen length (SL), pistil length (PL), Leaf blade length (BL), Leaf blade width (BW), blooming period (BBP), and flower color value (CCV) using heterosis analysis and a major gene plus polygene mixed genetic model. Ten phenotypic traits exhibited continuous, unimodal, and skewed distributions, with eight traits showing leptokurtic patterns (kurtosis > 0) and two traits showing platykurtic patterns (kurtosis < 0) across both populations, indicative of quantitative traits controlled by multiple genes. In Cross I, BL (-2.41) and BW (-2.04) displayed significant negative mid-parent heterosis, whereas BBP (7.99) and CCV (1.08) exhibited positive mid-parent heterosis. In Cross II, BL (-3.47) and BW (-1.61) showed significant hybrid depression, while BBP (6.54) and CCV (0.36) demonstrated significant mid-parent heterosis. Genetic analysis identified the optimal inheritance models as follows: Model 2MG-AD (two major genes with additive-dominance effects) for FS, FD, CL, and CW, with the first gene pair showing predominant additive effects (d_a_ = 1.81, 2.76, 1.31, and 0.70, respectively); Model 2MG-A (two major genes with additive effects) for SL, with the second gene pair contributing larger additive effects (d_b_ = 0.39); Model 2MG-ADI (two major genes with additive-dominance-epistatic effects) for PL and BL, with negative dominance effects (h_a_ = -0.55 and -1.15, respectively); Model 2MG-EA (two major genes with additive-equal effects) for BW and BBP, with additive effects of 0.65 and 10.86, respectively; and Model 1MG-A (one major gene with additive effects) for CCV, with an additive effect of 1.62. Except for BW, the major gene heritability for the remaining nine traits exceeded 88%, suggesting that these traits are under strong genetic control under the specific environmental conditions of this study. These findings provide valuable genetic information to support future breeding efforts in *Clematis*.

## Introduction

The genus *Clematis* L. (Ranunculaceae) comprises a vast group of predominantly woody vines. With approximately 355 species worldwide (including infraspecific taxa), the genus exhibits significant diversity, particularly in China, where about 147 species are native. Southwest China serves as a center of species diversity, while 32 species have been recorded in Zhejiang Province (Wang and Li, 2005; Li, 2021). Known as the “Queen of Vines,” *Clematis* is prized for its elegant form, rich coloration, and prolonged blooming period. Its ornamental value is derived primarily from floral traits, such as size, color, shape, and flowering habits (Shao et al., 2022). C*. tientaiensis* and *C. lanuginosa* are both classified as large-flowered *Clematis*, they bloom from April to June. The former features white sepals and purple-red anthers, whereas the latter has blue-purple sepals and reddish-brown anthers. Both species possess high ornamental value and represent native *Clematis* germplasm with considerable development potential.

Hybridization is a fundamental approach for germplasm innovation in ornamental plants. It facilitates the creation of novel phenotypic variations through genetic recombination and remains a primary method for modern cultivar development (Guo and Yang, 2025). Most target traits in ornamental breeding are quantitative in nature; their genetic architecture is typically governed by a few major genes acting in concert with multiple minor genes, a pattern described by the major gene plus polygene mixed genetic model (Pei et al., 2025). This model has been successfully applied to the genetic dissection of floral traits (e.g., color and form) in various ornamental species, including *Plumbago auriculata* (Chen et al., 2021), *Rosa chinensis* (Jiang et al., 2020), *Paeonia suffruticosa* (Zhang et al., 2018), and *Lilium* spp (Wang et al., 2021)., establishing a foundation for early selection in breeding programs (Pang et al., 2012; Yang et al., 2018; Xu et al., 2021; Chen et al., 2023). Recent advances in ornamental plant genomics have further revolutionized the genetic dissection of complex traits. High-quality genome assemblies and multi-omics analyses have been successfully employed to uncover the genetic architecture of floral traits in various ornamentals, including color formation in *Anthurium* (Lin et al., 2026), leaf color evolution in *Fittonia* (Wang L. et al., 2025), and color diversity in *Camellia* (Xiao et al., 2025). Among floral traits, the development of blue-flowered cultivars has long been a focal point in ornamental breeding, owing to the extreme rarity of true blue pigmentation in nature and its high commercial value (Tanaka et al., 2009; Noda, 2018). Blue flower coloration is primarily attributed to delphinidin-based anthocyanins, frequently modified through co-pigmentation and metal ion complexation, and has been extensively investigated in genera such as Gentiana, Delphinium, and Rosa (Yoshida et al., 2009; Sasaki and Nakayama, 2015). Although several blue- to purple-flowered cultivars exist within *Clematis*, the genetic basis underlying blue flower formation in this genus has yet to be elucidated. More broadly, a recent comprehensive review of *Clematis* highlighted that while significant progress has been made in understanding phylogenetic relationships and karyotype evolution, systematic genetic analyses of key ornamental traits—particularly flower color, size, and blooming period—remain scarce (Li et al., 2022; He et al., 2025). Research on *Clematis* hybrid breeding is still at an early stage. Existing studies have primarily focused on describing phenotypic variations, whereas systematic investigations of the genetic mechanisms governing key ornamental floral traits are lacking (Chen et al., 2017; Pang et al., 2024). As flower diameter, color, and flowering period are critical selection criteria in *Clematis* breeding, elucidating their inheritance patterns is essential for developing new cultivars and improving breeding efficiency.

The selection of *C. lanuginosa* and *C. tientaiensis* as parents was based on their complementary ornamental traits and phylogenetic relationships. *C. lanuginosa* (blue-purple-flowered) and *C. tientaiensis* (white-flowered) represent two distinct color types within the genus, providing an ideal system for investigating the inheritance of flower color, particularly the genetic determinants of blue pigmentation. Karyotype analysis has demonstrated that both species are diploid with highly symmetrical karyotypes (type 2A), indicating close phylogenetic affinity and high cross-compatibility (Li et al., 2022; He et al., 2025). Moreover, both species represent valuable native germplasm resources with considerable potential for ornamental breeding. Understanding the inheritance patterns of their floral traits is essential for the effective utilization of these native resources in cultivar development. Therefore, in this study, *C. lanuginosa* and *C. tientaiensis* were utilized as parents to construct reciprocal F_1_ populations. Using heterosis analysis and the major gene plus polygene mixed genetic model, we systematically investigated the inheritance patterns of ten floral and vegetative traits. The objectives of this study were to characterize the inheritance patterns of these traits, estimate major gene effects and heritability, and establish a foundation for future genetic studies and marker-assisted breeding in *Clematis*.

## Materials and methods

### Materials

**Figure 1:**
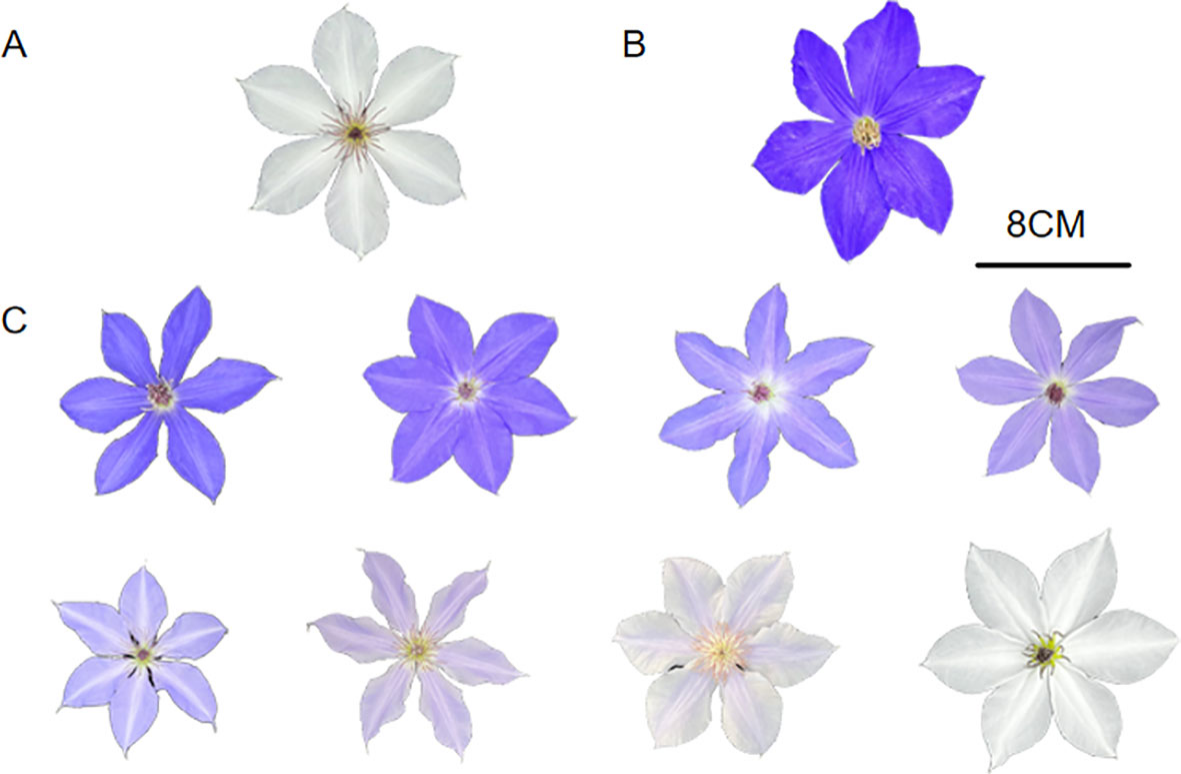
Morphology and color variation of the parental lines and representative F_1_ plants. **(A)***Clematis tientaiensis*; **(B)***Clematis lanuginosa*; **(C)** A representative individual from the F_1_ population.

Reciprocal hybridization experiments were conducted from May to June 2019 using *C. tientaiensis* (TT) and *C. lanuginosa* (MY) as parents. These crosses yielded two populations: Cross I (TT × MY) and Cross II (MY × TT).Prior to hybridization, all flower buds on the maternal parents were emasculated one day before anthesis to prevent self-pollination. The hybridization experiments were conducted in an open-air nursery under natural conditions. Immediately after artificial pollination, the inflorescences were covered with parchment bags to prevent contamination from unintended pollen sources. Bags were removed one week after pollination. Mature seeds were harvested in November 2019, sown, and the resulting seedlings were cultivated in pots at the *Clematis* Germplasm Resource Nursery of Zhejiang A&F University. The potting substrate consisted of a mixture of peat and perlite (1:1, v/v). Plants were maintained under 30% shade and subjected to standard horticultural management for irrigation and fertilization (Ingels, 2000). Given the perennial nature and extended juvenile phase characteristic of woody *Clematis* species, the F_1_ plants required several years of vegetative growth to reach reproductive maturity and produce flowers with stable, representative phenotypic traits. Following seed sowing in November 2019, the resulting seedlings were cultivated in pots and maintained under uniform nursery conditions at the *Clematis* Germplasm Resource Nursery. Standard horticultural management, including regular irrigation, fertilization, and weed control, was provided annually. By the spring of 2024 (the fifth growing season post-germination), the plants had entered a stable flowering phase, enabling the collection of robust and reliable phenotypic data for all ten floral and vegetative traits under investigation (**Figure 1**). A total of 120 flowers were pollinated for each cross combination (TT × MY and MY × TT). Mature seeds were harvested in November 2019, yielding 187 seeds for Cross I and 156 seeds for Cross II. After sowing and cultivation, 37 F_1_ individuals survived to flowering for Cross I and 24 for Cross II. All 37 individuals from Cross I were used for phenotypic data collection and subsequent mixed genetic analysis; Cross II was used only for phenotypic comparison due to its smaller sample size (n=24), which was insufficient for reliable genetic modeling.

### Criteria for determination of phenotypic traits

**Figure 2:**
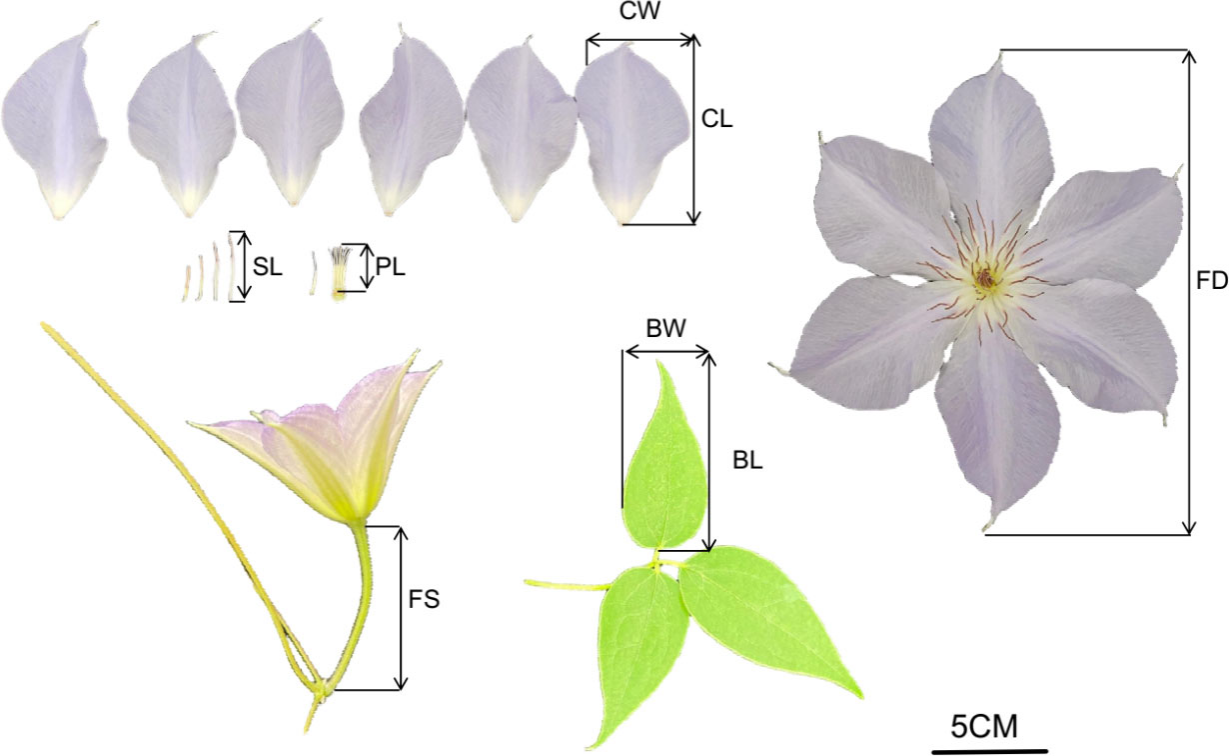
Schematic representation of phenotypic trait measurement methods in *Clematis* hybrids.

For each individual plant, five fully open flowers were randomly selected for the measurement of the following traits (**Figure 2**):

1. Flower stalk length (FS): Measured as the distance from the point of attachment on the stem to the base of the flower (unit: cm). Three measurements were taken per flower, and the mean value was calculated.
2. Flower diameter (FD): Defined as the maximum diameter measured across the center of symmetry (unit: cm). Three measurements were taken per flower, and the mean value was calculated.
3. Sepal length (CL) and sepal width (CW): Length was measured along the midrib of the sepal, and width was measured at the widest point perpendicular to the midrib (unit: cm).
4. Stamen length (SL) and pistil length (PL): Measured along the longitudinal axis of the stamen and pistil, respectively (unit: cm).
5. Leaf blade length (BL) and leaf blade width (BW): Length was measured along the midrib of the blade, and width was measured at the widest point perpendicular to the midrib (unit: cm).
6. Blooming period (BBP): Defined as the date of the first flower opening. Calendar dates were converted to numeric values representing the number of days from the onset of the flowering season (unit: days). April 15, 2024 (the earliest flowering date observed), was set as day 0. Subsequent dates were converted accordingly (e.g., April 17, 2024, was recorded as 2).
7. Flower color value (CCV): The color of sepals at full bloom was measured for all parental plants and F_1_ progeny (**Table 1**). A colorimeter was used to determine the CIELAB coordinates (L, a, and b values).

**Table 1:** Standard grading and scoring criteria for flower color in *Clematis* hybrids.

Grade	Flower color	L (Lightness)	a (Red-Green)	b (Yellow-Blue)	Score
I	White	>70.1	<1.63	>-5.6	0
II	light Blue-purple	60.7~82.90	1.11~5.67	-10.87~-6.31	1
III	light Blue	78.5~82.00	3.97~6.87	-16.93~-12.50	2
IV	Blue-purple	60.60~74.3	6.1~16.37	-30.57~-12.7	3
V	Blue	45.02~96.73	5.43~28.00	<-17.23	4

L, a, and b are the color coordinates obtained from a colorimeter. The L coordinate represents brightness; the a coordinate represents the red (positive)-to-green (negative) scale; the b coordinate represents the yellow (positive)-to-blue (negative) scale.

### Statistical analysis of phenotypic traits

Descriptive statistics for the F_1_ generation, including mean, standard deviation, range, and coefficient of variation (CV), were calculated. Frequency distributions were generated using Origin 2024 to visualize trait distribution patterns.

Heterosis Analysis and Significance TestingHeterosis was evaluated based on the collected phenotypic data using two metrics: mid-parent heterosis (Hm) and relative mid-parent heterosis (RHm) (Zhang et al., 2024). Mid-parent heterosis (Hm) was defined as the difference between the mean of the F_1_ generation (Fm) and the mid-parent value (MPV). Relative mid-parent heterosis (RHm) was calculated as the ratio of Hm to MPV (RHm =Hm/MPV).

Statistical significance of heterosis was assessed using a one-sample t-test comparing the F_1_ generation mean against the mid-parent value. This test was chosen because it directly evaluates whether the observed deviation from the mid-parent value is significantly different from zero, which is the null hypothesis expectation under no heterosis (Xue, 2025). Compared to alternative approaches such as ANOVA, the one-sample t-test provides a more straightforward and powerful test for detecting heterosis when comparing a single hybrid population against its parental midpoint. Analyses were performed using SPSS 27 (IBM Corp., Armonk, NY, USA).

### Statistical thresholds and model selection criteria

For all statistical tests, a significance level of α = 0.05 was used, with P < 0.05 considered statistically significant and P < 0.01 considered highly significant. Heterosis significance was assessed using one-sample t-tests comparing F_1_ means against mid-parent values.

For mixed genetic model analysis, candidate models were evaluated based on the Akaike Information Criterion (AIC). For each trait, the three models with the smallest AIC values were selected as candidate optimal models. The final optimal model was determined by combining AIC-based selection with goodness-of-fit tests, including uniformity tests (U_1_², U_2_², U_3_²), Smirnov test (nW²), and Kolmogorov test (Dn). A model was considered acceptable if all goodness-of-fit test P-values were > 0.05, indicating no significant deviation between the observed and expected distributions.

### Mixed genetic analysis

Due to the unsuitability of flower color value (CCV) data in Cross II for modeling, genetic analysis was restricted to the Cross I population. The exclusion of Cross II CCV data was primarily due to its limited sample size (n = 24) and skewed distribution. As shown in **Table 2**, the color distribution in Cross II was heavily skewed toward white-flowered phenotypes (58.33%), resulting in a non-normal distribution pattern that violates the assumptions of the mixed genetic model. Furthermore, previous studies have demonstrated that reliable parameter estimation in major gene plus polygene mixed models typically requires a minimum sample size of 30–50 individuals per population. The small sample size of Cross II would lead to unstable parameter estimates and inflated type II error rates. To avoid potential bias and ensure the robustness of our genetic inferences, we therefore restricted the genetic analysis to the Cross I population (n = 37), which exhibited a more balanced color distribution and met the minimum sample size requirements.

**Table 2:** Segregation of flower color scores in F_1_ from two crosses.

Combination	♀	♂	F_1_ generation quantity	Color distribution of F_1_ generation(%)				
				Blue	Blue-purple	light Blue	light Blue-purple	White
Cross I (TT×MY)	White	Blue	37	24.32	10.81	29.73	27.03	8.11
Cross II (MY×TT)	Blue	White	24	4.17	4.17	8.33	25.00	58.33

TT, *Clematis tientaiensis* (white); MY, *C. lanuginosa* (blue). See **Table 1** for color grading criteria.

Using the major gene plus polygene mixed genetic model for quantitative traits in a single generation, maximum likelihood values (MLV) were calculated for a set of candidate genetic models. The Akaike Information Criterion (AIC) was subsequently derived from the MLVs to identify the optimal model. The goodness-of-fit was verified using uniformity tests (U_1_², U_2_², U_3_²), the Smirnov test (nW²), and the Kolmogorov test (Dn). The model with the lowest AIC value that also passed the goodness-of-fit tests was selected as the optimal genetic model. Based on this model, the least squares method was employed to estimate genetic parameters, including major gene effects, variances, and heritability. Major gene heritability was calculated as *h_mg_^2^=σ_mg_^2^/σ_p_^2^*(where *h_mg_^2^* = major gene heritability; *σ_mg_^2^* = major gene variance; *σ_p_^2^* = phenotypic variance). Analyses were performed using the SEA (Segregation Analysis) software package provided by the State Key Laboratory of Crop Genetics and Germplasm Enhancement, Nanjing Agricultural University.

## Results and analysis

### Phenotypic variation in the F_1_ generation of *Clematis*

Statistical analysis of phenotypic segregation in the F_1_ generation (**Supplementary Table 1**) revealed that all ten traits exhibited varying degrees of variation. The coefficient of variation (CV) ranged from 14.92% to 65.13% in Cross I and from 6.63% to 62.60% in Cross II. Notably, the CVs for all ten traits in Cross I exceeded 14%, indicating substantial genetic variability. Both BBP and CCV displayed high levels of variation across the two populations, with CVs exceeding 29% for BBP and 60% for CCV. These findings suggest a high probability of generating novel phenotypes for flowering period and flower color—two critical ornamental traits—in *Clematis* hybrid breeding, with Cross I generally exhibiting higher mean values for most traits compared to Cross II, except for BW.

**Figure 3:**
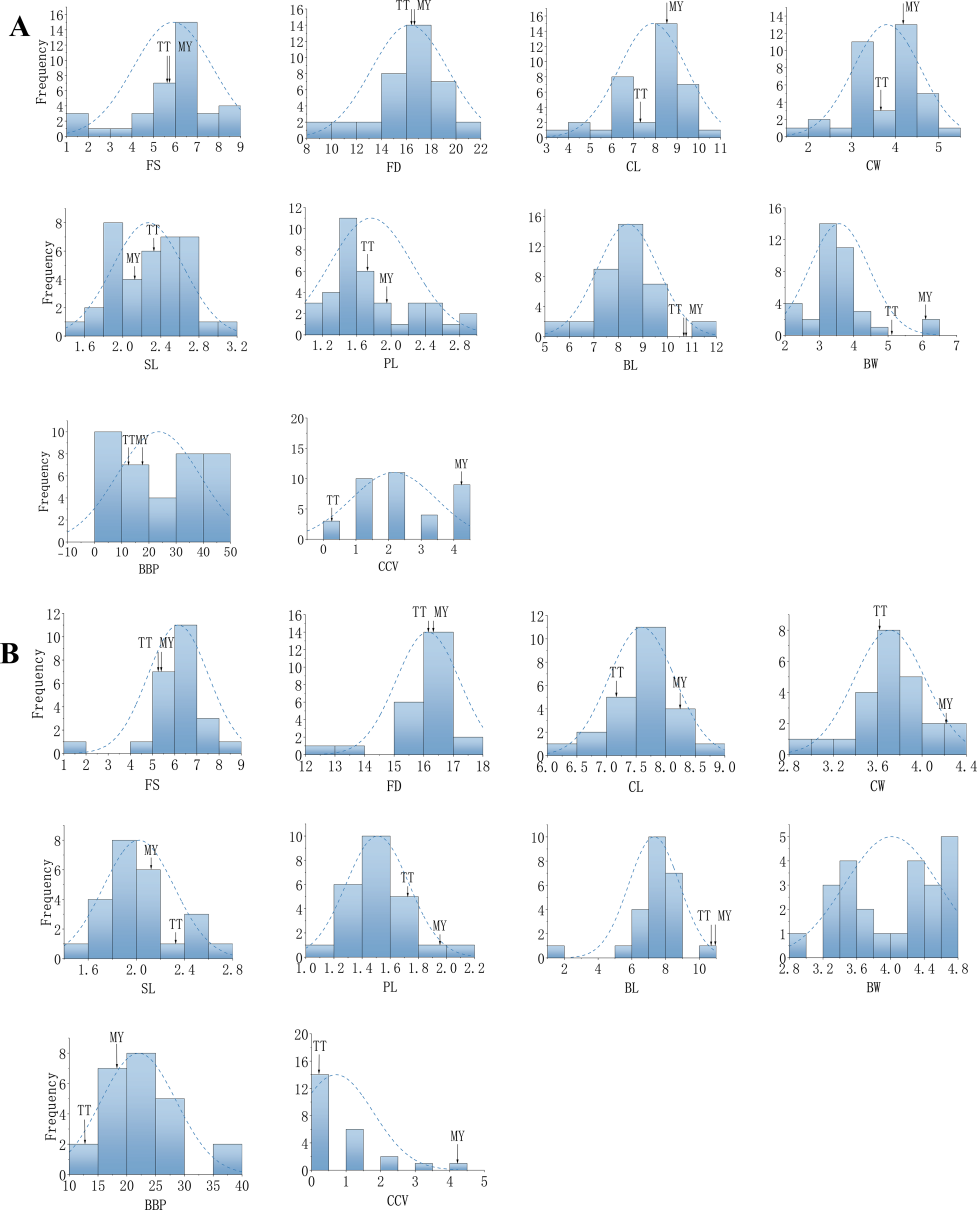
Frequency distribution of phenotypic traits in F_1_ populations. **(A)** Cross combination I (TT × MY); **(B)** Cross combination II (MY × TT). Detailed descriptive statistics are provided in **Supplementary Table 1**.

Further analysis of segregation patterns was conducted using skewness, kurtosis (**Supplementary Table 1**), and frequency distributions (**Figure 3**). In Cross I, FD, PL, and BBP displayed wide, continuous distributions; similarly, FD and BBP in Cross II exhibited continuous variation. With the exception of CCV, the traits in the F_1_ populations followed either normal or skewed continuous distributions. These patterns indicate that the measured characteristics are quantitative traits controlled by multiple genes, confirming their suitability for subsequent genetic analysis.

Kurtosis analysis revealed varying degrees of tailedness across traits and populations. In Cross I, six traits (FS, FD, CL, CW, BL, and CCV) exhibited leptokurtic distributions (kurtosis > 0), indicating heavier tails and a sharper peak than normal distribution, while four traits (SL, PL, BW, and BBP) showed platykurtic distributions (kurtosis < 0), suggesting lighter tails and a flatter peak. In Cross II, seven traits (FS, FD, CL, CW, PL, BL, and CCV) were leptokurtic, with particularly high kurtosis values for FS (7.24), FD (4.43), and BL (8.46), indicating strong central clustering; the remaining three traits (SL, BW, and BBP) were platykurtic. These kurtosis patterns, combined with the continuous distributions observed, confirm the quantitative nature of these traits and the presence of polygenic inheritance.

### Heterosis of phenotypic traits in the F_1_ generation of *Clematis*

As summarized in **Table 3**, nine of the ten phenotypic traits showed consistent heterosis patterns across both crosses, with SL being the exception. Specifically, BL and BW exhibited negative mid-parent heterosis (Cross I: -2.41 and -2.04; Cross II: -3.47 and -1.61), while BBP and CCV showed positive heterosis (Cross I: 7.99 and 1.08; Cross II: 6.54 and 0.36). FS also displayed positive heterosis in both crosses, along with transgressive segregation (**Figure 3**), indicating strong potential for genetic improvement. FD, CL, CW, and PL showed slightly negative or nonsignificant heterosis, suggesting that these traits are genetically conservative or regulated by polygenes with minor effects in the F_1_ generation, resulting in limited heterotic expression. SL, however, exhibited divergent heterosis trends between the two combinations. Transgressive segregation was observed for FS, CL, BL, and CCV in the F_1_ populations (**Figure 3**). Notably, only the mean values for FS and BBP exceeded those of the higher parent, indicating the presence of high-parent heterosis in both combinations. These findings provide a valuable basis for selecting individuals with longer flower stalks and extended flowering periods in breeding programs.

**Table 3:** Heterosis analysis of quantitative traits in F_1_ of two cross combinations.

(A) Cross Combination I (TT × MY).						
Traits	♀	♂	Mid-parent value	Mean value	Mid-parent heterosis (Hm)	Ratio of mid-parent heterosis (RHm)
FS	5.62 ± 0.18	5.72 ± 1.02	5.67	**5.89**	0.22	3.88
FD	16.43 ± 1.12	16.86 ± 1.05	16.65	16.27	-0.38	-2.28
CL	7.29 ± 0.55	8.54 ± 0.24	7.92	7.89	-0.03	-0.38
CW	3.63 ± 1.08	4.21 ± 1.88	3.92	3.81	-0.11	-2.81
SL	2.32 ± 0.87	2.13 ± 0.11	2.23	2.27	0.04	1.79
PL	1.71 ± 1.28	1.96 ± 0.76	1.84	1.77	-0.07	-3.80
BL	10.75 ± 1.88	10.82 ± 1.2	10.79	8.38	-2.41**	-22.34
BW	5.14 ± 1.29	6.09 ± 1.99	5.62	3.58	-2.04**	-36.30
BBP	13.00 ± 2.13	18.00 ± 1.66	15.5	**23.49**	7.99**	51.55
CCV	0.00 ± 0.00	4.00 ± 0.00	2.00	2.16	1.08**	54.00
(B) Cross Combination II (MY × TT)						
FS	5.72 ± 1.02	5.62 ± 0.18	5.67	**6.16**	0.49	8.64
FD	16.86 ± 1.05	16.43 ± 1.12	16.65	16.14	-0.51	-3.06
CL	8.54 ± 0.24	7.29 ± 0.55	7.92	7.60	-0.32	-4.04
CW	4.21 ± 1.88	3.63 ± 1.08	3.92	3.71	-0.21	-5.36
SL	2.13 ± 0.11	2.32 ± 0.87	2.23	2.02	-0.21	-9.42
PL	1.96 ± 0.76	1.71 ± 1.28	1.84	1.51	-0.33	-17.93
BL	10.82 ± 1.2	10.75 ± 1.88	10.79	7.32	-3.47**	-32.16
BW	6.09 ± 1.99	5.14 ± 1.29	5.62	4.01	-1.61**	-28.65
BBP	18.00 ± 1.66	13.00 ± 2.13	15.5	**22.04**	6.54**	42.19
CCV	4.00 ± 0.00	0.00 ± 0.00	2.00	0.71	0.36**	18.00

Traits abbreviations are as defined in **Supplementary Table 1**. MPV, (P♀ + P♂)/2; Hm, Mean(F_1_) - MPV; RHm, (Hm/MPV) × 100%. Data are presented as mean ± standard deviation for parental values. Bold F_1_ Mean indicates values exceeding the higher parent. * and ** indicate significant mid-parent heterosis at the 0.05 and 0.01 probability levels, respectively; the significance test for the F_1_ group’s intermediate parent advantage is based on the comparison between the average values of the target trait in F_1_ single plants and those of the intermediate parent.

### Suitability analysis of the optimal genetic model for phenotypic traits

Owing to the small population size of Cross Combination II, it was not suitable for constructing a mixed genetic model. Therefore, genetic analysis was performed using the mean values of 10 phenotypic traits of Cross Combination I, and the results are presented in **Table 4**. Based on the values of 10 quantitative phenotypic traits of the F_1_ generation, the maximum likelihood values (MLV) and Akaike information criterion (AIC) values were calculated via the mixed genetic model analysis method. The three models corresponding to the smallest AIC values were selected as candidate optimal models. For instance, the AIC values of models such as 1MG-A, 1MG-EAD and 1MG-NCD for CCV were relatively low, which were 108.1844, 128.8462 and 123.9318, respectively. Meanwhile, the goodness-of-fit of the candidate models was verified using the U_1_², U_2_², U_3_², nW², and Dn tests (**Table 5**). The screening results for each trait were as follows: the optimal genetic model for FS, FD, CL and CW was 2MG-AD, indicating that these traits were controlled by two pairs of major genes with additive-dominant effects; the optimal model for SL was 2MG-A, which implied the regulation by two pairs of major genes with additive effects; the optimal model for PL and BL was 2MG-ADI, suggesting the control by two pairs of major genes with additive-dominant-epistatic effects; the optimal model for BW and BBP was 2MG-EA, corresponding to the regulation by two pairs of major genes with additive-equivalent effects; the optimal model for CCV was 1MG-A, indicating the control by one pair of major genes with additive effects. The goodness-of-fit test statistics of the 10 main traits were not significant, which demonstrated that the selected models were consistent with the distribution of the segregating population, thus facilitating the acquisition of accurate genetic analysis results. Due to the limited population size of Cross II, this group was unsuitable for mixed genetic model analysis. Consequently, genetic analysis was restricted to the ten phenotypic traits assessed in the Cross I population (**Table 4**). Maximum likelihood values (MLV) and Akaike Information Criterion (AIC) values were calculated for each trait using the mixed genetic model method. For each trait, the three models with the lowest AIC values were identified as candidate optimal models. For example, regarding CCV, models 1MG-A, 1MG-EAD, and 1MG-NCD yielded the lowest AIC values of 108.18, 128.85, and 123.93, respectively. The goodness-of-fit for these candidate models was subsequently verified using uniformity (U_1_², U_2_², U_3_²), Smirnov (nW²), and Kolmogorov (Dn) tests (**Table 5**). The optimal genetic models identified for each trait are summarized as follows: FS, FD, CL, and CW: The optimal model was 2MG-AD, indicating control by two pairs of major genes with additive-dominance effects. SL: The optimal model was 2MG-A, implying regulation by two pairs of major genes with additive effects. PL and BL: The optimal model was 2MG-ADI, suggesting control by two pairs of major genes with additive-dominance-epistatic effects. BW and BBP: The optimal model was 2MG-EA, corresponding to regulation by two pairs of major genes with additive-equal effects. CCV: The optimal model was 1MG-A, indicating control by a single pair of major genes with additive effects. The goodness-of-fit test statistics for all ten traits were nonsignificant (P>0.05), confirming that the selected models adequately described the distribution of the segregating population. This validation ensures the reliability of the subsequent genetic parameter estimates.

**Table 4:** AIC values of candidate genetic models for ten quantitative traits in F_1_.

Model	Model in plication	FS	FD	CL	CW	SL	PL	BL	BW	BBP	CCV
A→0	0MG	152.2676	191.6182	140.9555	89.4370	35.4785	54.3644	124.2795	97.5961	309.8538	127.5300
A→1	1MG-AD	137.4497	188.6076	136.9392	88.6430	35.9086	41.2959	123.6346	83.1044	299.7326	_
A→2	1MG-A	137.6534	187.9130	136.1539	86.7984	35.6593	44.9707	121.7802	82.1515	298.8566	**108.1844**
A→3	1MG-EAD	145.8518	189.5646	138.6942	90.3737	36.4108	46.6024	125.1881	90.7434	303.7098	128.8462
A→4	1MG-NCD	156.2557	195.6066	144.9438	93.4258	39.4716	44.4605	128.1312	80.1969	313.7134	123.9318
B→1	2MG-ADI	142.7209	190.9782	141.8398	81.6005	47.8513	**28.0620**	**105.6051**	84.3078	301.8433	**_**
B→2	2MG-AD	**129.8317**	**148.8541**	**116.5074**	**66.7808**	23.7669	29.3600	121.8064	67.5052	296.5647	-473.2748
B→3	2MG-A	138.7366	_	_	81.1547	**17.3067**	42.3497	123.9451	77.7502	298.2258	_
B→4	2MG-EA	138.4844	177.4114	129.0051	79.5469	21.7543	42.4474	121.8696	**65.8350**	**294.9343**	_
B→5	2MG-CD	143.2422	185.4068	132.5001	83.4892	38.8502	58.3531	128.2702	101.5840	299.5667	131.5235
B→6	2MG-EAD	147.5679	183.4062	130.4993	81.4892	37.3991	56.3531	126.2702	99.5840	297.5658	129.5236

MG denotes the major gene pattern; A represents additive effects, D denotes dominant effects; E indicates equal effects; N denotes negative effects, C denotes completion, and I denotes ineffectiveness. Bold values indicate the optimal model for each trait based on the smallest AIC.

**Table 5:** Adaptability test results of F_1_ representative quantitative traits selection model.

Traits	Optimal Model	U_1_^2^	P(U_1_^2^)	U_2_^2^	P(U_2_^2^)	U_3_^2^	P(U_3_^2^)	_n_W^2^	P_(n_W^2^)	D_n_	P(D_n_)
FS	2MG-AD	0.0052	0.9424	0.0019	0.9655	0.0114	0.9149	0.0109	1.0000	0.0476	1.0000
FD	2MG-AD	0.0270	0.8694	0.0375	0.8464	0.0192	0.8899	0.0111	1.0000	0.0533	0.9997
CL	2MG-AD	0.0051	0.9429	0.0148	0.9032	0.0438	0.8341	0.0130	0.9999	0.0545	0.9996
CW	2MG-AD	0.0074	0.9313	0.0126	0.9106	0.0133	0.9083	0.0152	0.9996	0.0566	0.9993
SL	2MG-A	0.5842	0.4447	0.5613	0.4537	0.0013	0.9709	0.0591	0.8219	0.0765	0.9703
PL	2MG-ADI	0.0021	0.9632	0.0009	0.9763	0.0036	0.9524	0.0109	1.0000	0.0486	1.0000
BL	2MG-ADI	0.0000	0.9965	0.0000	0.9997	0.0002	0.9876	0.0144	0.9997	0.0521	0.9998
BW	2MG-EA	0.0869	0.7681	0.3003	0.5837	1.1025	0.2937	0.0952	0.6208	0.1273	0.5444
BBP	2MG-EA	0.0041	0.9487	0.0067	0.9347	0.3329	0.5640	0.0323	0.9682	0.0800	0.9568
CCV	1MG-A	0.0659	0.7974	0.2004	0.6544	0.6344	0.4258	0.2809	0.1599	0.2038	0.0798

U_1_^2^, U_2_^2^, U_3_^2^, _n_W^2^, and D_n_ are the five adaptability test statistics, and P is the corresponding probability value.P is the corresponding probability value.

### Estimation of genetic parameters for main floral traits under the optimal fitted genetic models

The genetic parameters estimated from the optimal models for the ten quantitative phenotypic traits in the F_1_ generation are summarized in **Table 6**. For FS, FD, CL, PL, and BL, the additive effect values (d_a_, d_b_) for both pairs of major genes were positive. Notably, the additive effect of the first major gene pair exceeded that of the second, indicating that the additive genetic variance for these five traits was primarily governed by the first gene pair. Conversely, for SL, the additive effect was dominated by the second gene pair (0.39). Regarding dominance effects (h_a_, h_b_), FD exhibited positive values for both gene pairs, with the first pair exerting a predominant effect (2.07). In contrast, PL and BL displayed negative dominance effects, which were also primarily driven by the first gene pair (-0.55 and -1.15, respectively). With the exception of BW, the major gene heritability for the remaining nine traits exceeded 88% under the uniform conditions of this study, indicating strong genetic control of these traits.

**Table 6:** Estimation of genetic parameters of quantitative traits of F_1_ under the optimal model.

Traits	M	d_a_	d_b_	h_a_	h_b_	i	j_ab_	j_ba_	l	σ^2p^	σ^2mg^	h^2mg^(%)
FS	5.1242	1.8116	0.7569	2.4378	-1.4675	_	_	_	_	0.3797	2.9284	88.5223
FD	14.4980	2.7583	2.3427	2.0698	0.5994	_	_	_	_	0.0981	9.4842	98.9764
CL	7.7848	1.3077	0.3916	0.9357	-0.7699	_	_	_	_	0.0089	2.4278	99.6342
CW	4.0485	0.7029	-0.0084	0.2285	-0.7862	_	_	_	_	0.0002	0.6053	99.9749
SL	2.1516	0.0900	0.3883	_	_	_	_	_	_	0.0002	0.1407	99.8629
PL	1.9161	0.6064	0.2635	-0.5503	-0.0255	0.0540	-0.0703	0.1895	0.3040	0.0017	0.2330	99.2863
BL	8.5127	1.7789	1.1911	-1.1532	-0.2200	0.0873	-0.5562	0.1096	1.0385	0.0131	1.5396	99.1581
BW	3.7344	0.6464	_	_	_	_	_	_	_	1.7634	0.0000	0.0000
BBP	23.2884	10.8551	_	_	_	_	_	_	_	2.4079	231.6267	98.9712
CCV	2.2454	1.6205	_	_	_	_	_	_	_	0.0180	1.6772	98.9390

m denotes the population mean; d_a_ represents the additive effect of the first pair of major genes; d_b_ denotes the additive effect of the second pair of major genes; h_a_ indicates the dominant effect of the first pair of major genes; h_b_ denotes the dominant effect of the second pair of major genes; i represents the additive-additive interaction; j_ab_ denotes the additive-dominant interaction; j_ba_ indicates the dominant-additive interaction; l denotes the dominant-dominant interaction; σ^2p^ denotes phenotypic variance;σ^2mg^ denotes major gene variance; h^2mg^ denotes major gene heritability.

## Discussion

Heterosis serves as a fundamental strategy for trait improvement in ornamental plants (Zhang et al., 2022; Jiang et al., 2023; Wang, 2023; Sun YT. et al., 2024; Wan et al., 2024), with its magnitude largely contingent upon the genetic divergence between parents (Qi, 2017; Liu et al., 2024; Mu et al., 2024; Sun WH. et al., 2024). In this study, wild *Clematis* species exhibiting distinct differences in flower color were selected as parents, establishing a robust foundation for analyzing heterosis in the F_1_ generation. Our results demonstrate significant positive heterosis for flower stalk length (FS), blooming period (BBP), and flower color value (CCV). These findings align with previous studies on *Plumbago auriculata* (Chen et al., 2021), *Paeonia suffruticosa* (Zhang et al., 2018), *Lilium* hybrids (Wang et al., 2021), and *Phalaenopsis* (Wang RH. et al., 2025; Wang YC. et al., 2025), suggesting that hybridization is an effective means of improving these traits. Conversely, most floral and leaf morphological traits (e.g., FD, CL, CW) exhibited significant negative mid-parent heterosis, indicating a tendency toward miniaturization in the hybrid progeny. Similar trends have been observed in other taxa, such as double-flowered crabapple (Li et al., 2025) and *Hemerocallis fulva* (Bai et al., 2023), Notably, this “miniaturization” effect has been recognized as a valuable breeding outcome in other ornamental genera, facilitating the development of compact, delicate cultivars suitable for container gardening and urban landscapes (Chen et al., 2022; Li et al., 2024). Thus, our findings provide both a novel phenotypic basis and theoretical support for the directional breeding of “small-flowered and delicate” *Clematis* lines. Furthermore, transgressive segregation for FS and BBP was observed in the F_1_ population, indicative of complementary gene action. This phenomenon, often attributed to the recombination of favorable alleles from divergent parents in interspecific hybrids, has been successfully exploited in other perennial ornamentals to achieve trait breakthroughs (Liu et al., 2022; Yang et al., 2023). Consequently, elite individuals with elongated flower stalks and extended blooming periods can be directly selected from this population, offering significant implications for parent selection strategies tailored to specific ornamental breeding objectives.

Based on the major gene plus polygene mixed genetic model, this study elucidated the optimal inheritance mechanisms governing each trait. Key ornamental traits, including flower stalk length (FS), flower diameter (FD), and sepal size (CL, CW), were all controlled by two pairs of major genes with additive-dominance effects (2MG-AD model). This suggests a relatively complex genetic architecture where dominance effects play a pivotal role in shaping the F_1_ phenotype. These findings align with previous reports on *Paeonia suffruticosa* (Yang et al., 2024), double-flowered crabapple, and *Chrysanthemum morifolium* (Liang, 2023). In contrast, leaf width (BW) and blooming period (BBP) conformed to the two-pair additive-equal major gene model (2MG-EA model), and their genetic patterns were consistent with those of Korla fragrant pear (Xue, 2025). The inheritance of flower color value (CCV) was the simplest, conforming to a single major gene model with additive effects (1MG-A model). This implies that flower color performance in the progeny is highly predictable, facilitating directional improvement through strategic parent selection, similar to results observed in pink-flowered *Rosa chinensis* (Jiang et al., 2020). Heritability analysis revealed that, Except for BW, the major gene heritability for the other nine traits exceeded 88%, with FD, CL, CW, SL, PL, BBP, and CCV surpassing 90%. Such high heritability, estimated under uniform nursery conditions, indicates that these traits are predominantly genetically controlled. However, as our study was conducted in a single environment, the stability of these traits across different environmental conditions requires further validation through multi-environment trials. Consequently, phenotypic selection in early generations (e.g., the F_1_ generation) is highly reliable and can significantly enhance breeding efficiency. These findings provide critical parameter support for early selection strategies in *Clematis* breeding.

A limitation of this study is that genetic analysis was restricted to a single F_1_ generation. Due to the self-incompatibility and high heterozygosity inherent in *Clematis* species, developing stable inbred lines is challenging. Consequently, the current model has limited power to distinguish minor polygenic effects from environmental variance. To achieve a more precise dissection of polygenic effects, future research should focus on constructing complex populations, such as backcross (BC) or F2 generations, combined with multi-year and multi-environment phenotypic evaluation (Zhou, 2023). Furthermore, the genetic models and major gene effects identified herein provide fundamental insights into the inheritance of key ornamental traits, offering a theoretical basis for parental selection and early-generation selection in *Clematis* breeding programs. These findings also lay the groundwork for future molecular studies, including quantitative trait locus (QTL) mapping and marker-assisted selection (MAS), once appropriate molecular tools and mapping populations become available (Fan, 2021; Wang, 2022; Qin, 2023).

Additionally, the exclusion of Cross II from genetic modeling may introduce potential bias in interpreting the inheritance patterns across reciprocal crosses. The strong maternal effect observed in Cross II, where the blue-flowered mother (MY) produced predominantly white-flowered progeny (58.33%), suggests that cytoplasmic inheritance or epigenetic modifications might play a role in flower color determination. Future studies incorporating reciprocal crosses with larger population sizes and molecular markers are needed to dissect the underlying mechanisms of these maternal effects.

Consequently, the exclusion of this dataset reduces the statistical power and generalizability of our findings, particularly regarding the inheritance patterns of flower color across different cross directions. While the results from Cross I (n=37) provide robust estimates under the 2MG-AD and 1MG-A models, the inability to model Cross II limits our understanding of potential maternal effects and genotype-by-environment interactions that may influence trait expression. Future studies with larger reciprocal populations (≥50 individuals per cross) are necessary to validate these findings and fully capture the genetic architecture of these traits.

While most floral traits showed high heritability (>88%), genotype-by-environment interactions may still influence trait expression in practical breeding. Temperature, light, and soil conditions can affect phenotypes, especially for BBP and CCV, which exhibited high CVs (29.63–65.13%). Although our plants were grown under uniform conditions, G×E interactions become important when breeding for broader adaptation. Multi-environment trials are needed to validate genetic models. It is important to note that our conclusion regarding the stability of these traits is based solely on observations from a single environment. Without multi-environment validation, statements about “minimal environmental influence” remain speculative. Therefore, while our results demonstrate strong genetic control under the evaluated conditions, caution should be exercised when extrapolating these findings to other environments. Future studies across multiple locations and years are essential to confirm the stability of these traits and to quantify the magnitude of genotype-by-environment interactions.

Based on our genetic findings, we propose a three-stage breeding strategy for developing novel *Clematis* cultivars:

### Stage 1

Parental selection and cross design (Year 1).For blue-flowered cultivars: Use *C. lanuginosa* as the maternal parent to leverage its strong maternal effect (58.33% blue-derived progeny in Cross II).For white-flowered cultivars: Use *C. tientaiensis* as the maternal parent to maximize white progeny production. For flower size improvement: Select parents with complementary additive effects for FD and CL based on the 2MG-AD model parameters (d_a_=2.76 for FD).

### Stage 2

Early-generation screening (Year 5-6, after juvenile phase). Screen F_1_ populations at first flowering for: Flower color: Select individuals with scores 0–1 for white series, 3–4 for blue series (predictable from 1MG-A model). Flower diameter: Retain individuals with FD > 16.5 cm (exploiting positive dominance effect of 2.07). Blooming period: Select early-flowering individuals (BBP < 20 days) for extended season cultivars. Minimum population size: Maintain at least 50 F_1_ individuals per cross to ensure recovery of rare recombinants

### Stage 3

Multi-environment validation and cultivar release (Year 7-8). Evaluate selected elite clones across 3–4 representative locations in Zhejiang Province (mountainous, coastal, and urban sites). Conduct 2-year stability trials for BBP and CCV, which showed high environmental sensitivity (CV > 60%). For BW (zero heritability), rely on clonal replication rather than single-plant selection; select based on 3-year performance means.

This breeding scheme integrates our genetic parameters into a time-bound roadmap, enabling efficient development of *Clematis* cultivars with targeted flower colors, improved flower size, and stable performance across environments. In conclusion, this study represents the first systematic investigation into the inheritance patterns of key floral traits in F_1_ derived from *C. tientaiensis* × *C. lanuginosa*. We elucidated the direction of heterosis, identified optimal genetic models, and confirmed the high heritability of these traits. These findings provide a theoretical basis and practical guidelines for parent selection and early-generation selection in *Clematis* hybrid breeding. Moreover, this work establishes a solid foundation for future molecular mapping, QTL analysis, and marker-assisted breeding. Finally, the phenotypically diverse individuals generated in this study serve as valuable germplasm for future breeding programs.

## Conclusion

In this study, a systematic analysis was conducted on ten floral and vegetative traits in F_1_ derived from the cross between *C. tientaiensis* and *C. lanuginosa*. All investigated traits were quantitative in nature. With the exception of flower color value (CCV), which was controlled by a single major gene pair, the remaining traits were governed by two pairs of major genes, fitting various mixed genetic models, including 2MG-AD, 2MG-A, 2MG-ADI, and 2MG-EA. Heterosis analysis indicated that the F_1_ generation generally exhibited negative heterosis for floral and leaf size traits. In contrast, significant positive heterosis was observed for flower stalk length (FS), blooming period (BBP), and CCV. Notably, transgressive segregation was detected for FS and BBP, providing clear opportunities for breeding novel cultivars characterized by smaller flowers, extended blooming periods, and superior coloration. Genetic parameter estimation revealed that, except for leaf width (BW), the major gene heritability for the other nine traits exceeded 88%, indicating strong genetic control under the evaluated conditions. This supports the feasibility of phenotypic selection in early generations. However, as these findings were obtained from a single environment, conclusions about trait stability across diverse environments remain tentative. Multi-environment trials are therefore necessary to confirm the stability of these traits and to validate the broader applicability of our findings. Therefore, future studies with larger reciprocal populations and multi-environment evaluations are recommended to further validate and extend our findings. To our knowledge, this is the first study to elucidate the inheritance patterns underlying key ornamental traits in *Clematis*. These findings provide direct theoretical guidance for parent selection and early-generation selection in hybridization programs, establishing a critical foundation for future genetic studies and breeding applications in *Clematis*.

## Data Availability

The raw data supporting the conclusions of this article will be made available by the authors, without undue reservation.
